# Once daily aerosolised tobramycin in adult patients with cystic fibrosis in the management of *Pseudomonas aeruginosa* chronic infection

**DOI:** 10.1186/s40248-016-0083-y

**Published:** 2017-02-07

**Authors:** Marco Mantero, Andrea Gramegna, Giovanna Pizzamiglio, Alice D’Adda, Paolo Tarsia, Francesco Blasi

**Affiliations:** Department Pathophysiology and Transplantation, University of Milan, IRCCS Fondazione Ca’ Granda Ospedale Maggiore Policlinico, via Francesco Sforza 35, Milan, 20122 Italy

**Keywords:** *P. aeruginosa* chronic infection, Tobramycin, Aerosolised antibiotic therapy, Cystic fibrosis

## Abstract

It is estimated that about 60–70% of Cystic Fibrosis patients develop *Pseudomonas aeruginosa* chronic infection, with progressive loss of lung function, as well as increased antibiotic resistance and mortality.

The current strategy is to maintain lung function by chronic suppressive antipseudomonas antibiotic therapy. Tobramycin inhalation solution was the first approved aerosolised antibiotic to be used against *P. aeruginosa*; inhalatory tobramycin frequency of administration is twice daily and inhalation time is estimated to be 15 to 20 min.

From the pharmacokinetic point of view, aminoglycosides are dose-dependent antibiotics and therefore once-daily dosing intravenous regimens have shown to be superior to the conventional multiple daily dosing. Therefore, there is no pharmacological reason to prefer the b.i.d administration as it is usually performed in current clinical practice.

Should this be confirmed also for inhalatory route, the use of once-daily dosed aerosolized tobramycin could be an important step in making treatment burden easier in CF patients.

The aim of this proof of concept study was to explore the effectiveness of treatment with once daily inhaled tobramycin in reducing *P. aeruginos* a density in sputum of chronically infected patients.

It is estimated that during their lifetime about 60–70% of Cystic Fibrosis patients develop *P. aeruginosa* chronic infection, with progressive loss of lung function, as well as increased morbidity and mortality [1]. In addition, the massive use of antibiotics during bronchial exacerbations is associated with the selection of antibiotic resistance [2].

Commonly, a period of intermittent colonization by *P. aeruginosa* precedes the establishment of chronic infection and, in this window, efforts are made to obtain bacterial eradication by means of standardized antibiotic strategies.

Once infection is established, the current strategy is to maintain lung function by chronic suppressive antipseudomonas antibiotic therapy. Alongside the more traditional routes of administration (oral or IV), aerosol delivery has become a notable complementary strategy [3, 4].

Tobramycin inhalation solution (TIS) was the first approved aerosolised antibiotic to be used against *P. aeruginosa* (FDA, 1998). TIS frequency of administration is twice daily and inhalation time is estimated to be 15 to 20 min (excluding time for maintenance and disinfection of the nebulizer). Given the heavy burden of care in CF, attempts to reduce the time of administration have been discussed and a great number of studies addressing new delivery strategies have been reported [5, 6].

From the pharmacokinetic point of view, aminoglycosides are dose-dependent antibiotics and therefore once-daily dosing regimens have shown to be superior to the conventional multiple daily dosing [7]. Therefore, there is no pharmacological reason to prefer the b.i.d administration as it is usually performed in current clinical practice. Pharmacokinetic data on sputum concentration of tobramycin following aerosol administration are inconclusive [8] and recent data seems to indicate the possible efficacy of using once daily tobramycin in reducing *P. aeruginosa* bacterial load in chronic sinusitis in CF [9]. Should this be confirmed, the use of once-daily dosed aerosolized tobramycin could be an important step in making treatment burden easier in CF patients.

The aim of this proof of concept study was to explore the effectiveness of treatment with once daily inhaled tobramycin in reducing *P. aeruginosa* density in sputum of chronically infected patients.

A once daily dose regimen of 300 mg aerosolised tobramycin [Bramitob ®, Chiesi Farmaceutici] was administered for one month in adult CF patients with chronic *P. aeruginosa* infection, after a 4-week wash-out period of antispeudomonal therapy.

Patients were evaluated at T1, at the end of wash-out period, at T2, after 7 days of treatment, at T3, after 21 days of treatment and at T4, at the end of the 28-day treatment period.

The following parameters were analysed:

- Maximum bacterial load in sputum. (Max load)
- Mean bacterial load in sputum. (Mean load).
- Tobramycin-resistant *P. aeruginosa* strains (n°).
- Tobramycin-resistant *P. aeruginosa* strains (%).
- change in daily sputum amount.
- change in Forced Expiratory Volume in the first second (FEV_1_).
- change of systemic inflammatory markers, C-Reactive Proteine (CRP) and Procalcitonin (PCT).
- change in serum cytokines profile; Interleukin 2; (IL2), Interleukin 4 (IL4); Interleukin 6 (IL6); Interleukin 8 (IL8); Interleukin 10 (IL10); Vascular Endothelial Growth Factor (VEGF); Interferon Gamma (INFγ); Tumor Necrosis Factor Alfa (TNFα); Interleukin 1a (IL1a); Interleukin 1b (IL1b); Monocyte Chemoattractant Protein 1 (MCP1); Epidermal growth factor (EGF).

A comparison between data obtained at basal time (T1) and at T2, T3 and T4 was performed.

A total of 12 patients [7 males/2 females, mean age (SD) 35.1 (8.1) years] with chronic *P. aeruginosa* infection were enrolled. Nine patients completed the study, 2 were excluded from the study because sputum culture tests resulted negative for *P aeruginosa* before the first dose of tobramycin, and 1 patient withdrew consent.

No statistically significant differences in the Max and Mean bacterial load expressed as median and inter quartile range (IQR) at the different time points, were found; respectively 5.0 (4.5–5.5) log 10 CFU at T1, 5.0 (4.5–5–5) log 10 CFU at T2, 5.0 (5.0–6.0) log 10 CFU at T3 and 6.0 (5.0–6.0) log 10 CFU at T4; p (ANOVA) = 0,27 and 4.7 (3.9–4.9) log 10 CFU at T1, 4.7 (4.0–5.0) log 10 CFU at T2, 4.3 (4.3–5.3) log 10 CFU at T3 and 5.7 (3.7–6.0) log 10 CFU at T4 p (ANOVA) = 0.50. No difference in the mean (IQR) percent of predicted FEV_1_ at the different time points was found, 64 (60.50–80.00)% at T1 68.00 (59.00–79.50)% at T2, 69.00 (58.50–83.50)% at T3 and 67 (57.00–77.50)% at T4 p ANOVA = 0.97. Despite this, no increase in the number of resistant bacterial strains was registered and no difference in the mean (IQR) sputum 24-h production at the different time points were found, 25 (11.25–27.00) ml/day at T1, 20.00 (8.70–27.50) ml/day at T2, 15.00 (11.25–25.00) ml/day at T3 and 25.00 (11.25–30.00) ml/day at T4 p ANOVA = 0.93.

No difference in inflammatory markers was found, CRP expressed as median and IQR, at the different time points were 0.29 (0.21–0.44) mg/dl at T1, 0.39 (0.25–0.83) mg/dl at T2, 0.31 (0.25–0.56) mg/dl at T3 and 0.32 (0.20–1.36) mg/dl at T4 p ANOVA 0.86; PCT expressed as median and IQR was 0.05 (0.03–0.06) μg/ml at T1, 0.05 (0.04–0.06) μg/ml at T2, 0.31 (0.25–0.56) μg/ml at T3 and 0.32 (0.20–1.36) μg/ml at T4 p ANOVA = 0.86.

No difference in the cytokine profile was found at the different time points.

The analysis of the mean bacterial load showed reductions in some patients, but not in others, see Fig. 1.

**Fig. 1:**
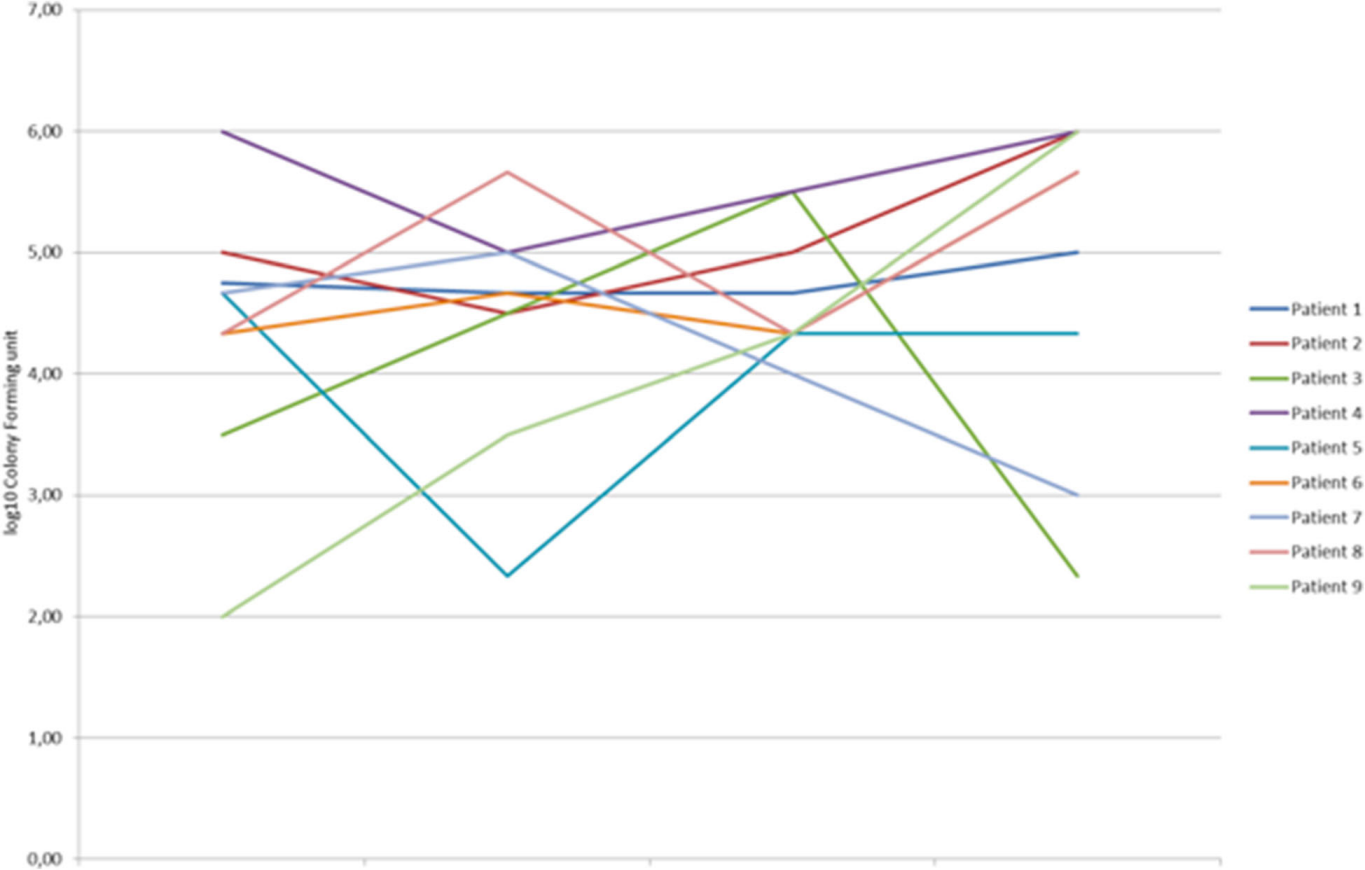
Mean bacterial load trend for each patient. T1: end of wash-out period; T2: 7 days after the beginning of treatment; T3: 21 days after the beginning of treatment; T4: end of the 28-day treatment

The main finding is that none of the selected outcomes (lung function, bacterial load, inflammation and cytokine profile) significantly changed during and at the end of the study, but some response may be observed in selected patients.

## Conclusion

These results may be related to the small sample size, but the lack of any positive trend suggests that the use of 300 mg of once daily areosolised tobramycin is insufficient in treating the majority of CF patients with chronic *P aeruginosa* infection. However, individual patients did benefit from once daily aerosolized tobramycin. Moreover, no increase in inflammatory and cytokine profile was found and no evidence of resistant strain selection emerged showing that in all patients some form of control of infection and inflammation was attained.

This leads us to conclude that it would be interesting to further explore the use of tobramycin with a phase II study with a dose-finding design and to confirm the existence of patients who are responders to once daily aerosolised tobramycin.

## Abbreviations

ANOVA: Analysis of variance
CF: Cystic fibrosis
CFU: Colony forming units
CRP: C-Reactive protein
EGF: Epidermal growth factor
FEV1: Forced expiratory volume in 1 s
IL: Interleukin
INFγ: Interferon gamma
IQR: Inter-quantile ranges
MCP1: Monocyte chemoattractant protein 1
PCT: Procalcitonin
TIS: Tobramycin inhalation solution
TNFα: Tumor necrosis factor alfa
VEGF: Vascular endothelial growth factor
